# Exploring adolescents’ ability to appraise health information: validation of the self-report short-form Critical Health Literacy Questionnaire

**DOI:** 10.3389/fpubh.2026.1881570

**Published:** 2026-07-09

**Authors:** Anders L. Hage Haugen, Kirsti Riiser

**Affiliations:** 1 Department of Primary and Secondary Teacher Education, Faculty of Teacher Education and International Studies, Oslo, Norway; 2 Department of Rehabilitation Science and Health Technology, Faculty of Health Sciences, Oslo Metropolitan University, Oslo, Norway; 3 Norwegian Institute of Public Health, Child and Adolescent Health Promotion Services, Levanger, Norway

**Keywords:** adolescent, critical health literacy, health literacy, health promotion, information appraisal, psychometrics, school, validation

## Abstract

**Introduction:**

Adolescents’ ability to evaluate health information is an increasingly important area in public health and health promotion; however, there is a scarcity of brief, psychometrically sound self-report tools focused on adolescents’ information appraisal abilities. In this study, we aim to validate a unidimensional short form of the Critical Health Literacy for Adolescents Questionnaire (CHLA-QA) domain A and to examine its convergent and known-groups validity.

**Methods:**

We conducted a cross-sectional survey among Norwegian students (*N* = 522; response rate 33%) aged 13–15 years old. We employed stepwise confirmatory factor analysis (CFA) to compare three models. Known-groups validity was assessed by examining the distribution of CHLA-QA across variables: sex, grade level, parents’ education, books at home, and generic health literacy (HLSAC), with analyses based on the Kruskal-Wallis test, the Wilcoxon rank-sum test, and effect sizes (ε^2^, r). We assessed convergent validity through Spearman correlations with HLSAC, self-rated health, HRQoL, and academic achievement.

**Results:**

Analysis yielded a five-item solution with acceptable fit (CFI = 0.98; RMSEA = 0.097 [0.065–0.132]; SRMR = 0.037, uSRMR/2 = 0.055), strong standardized loadings (0.582–0.839), and satisfactory reliability (*α* = 0.79). Known-groups analyses showed higher scores among girls than boys (*p* < 0.001; r = 0.169), a small grade effect (ε^2^ = 0.013; grade 9 > grades 8 and 10), and a socioeconomic gradient was found, with higher scores associated with parents’ education (r = 0.111) and number of books at home (ε^2^ = 0.024). HLSAC increased stepwise across low, moderate, and high groups based on the CHLA-QA scale (ε^2^ = 0.132). Convergent validity was supported by moderate correlations with HLSAC (*ρ* = 0.461), self-rated health (ρ = 0.316), and HRQoL (ρ = 0.346). Correlations with academic achievement were weak but significant for five of six school subjects. Higher scores were correlated with asking parents for help regarding health information (*ρ* = 0.233).

**Conclusion:**

This study advances the measurement of adolescents’ CHL by developing and evaluating a brief, unidimensional instrument for assessment of information appraisal (CHLA-QA). The scale showed strong validity and acceptable reliability. Future studies in independent samples are warranted to further establish validity and measurement invariance across relevant populations.

## 1 Introduction

Health literacy is a crucial element of public health, providing a foundation for both individual and community empowerment. It is understood as a continuum of social practices that include functional, interactive, and critical health literacy (1, 2). In recent years, critical health literacy (CHL) has emerged as a distinct area of interest within the field of health education and promotion (3–9). CHL builds on the concept of critical literacy, which focuses on the skills needed to analyze, understand, and effectively use information (1). These skills are crucial for empowering individuals to make informed decisions about their health. CHL goes beyond simply evaluating health-related information; it entails a deeper understanding of the social, cultural, and political contexts that shape health information (4, 9). By fostering CHL competencies, individuals are better equipped to navigate the complexities of health messages, advocate for their own health needs (5), and engage in meaningful dialogue with healthcare providers. Ultimately, enhancing CHL is essential for promoting autonomy and informed decision-making in an increasingly complex health information landscape.

The rise of misinformation and disinformation, often fueled by commercial interests, makes it increasingly difficult to distinguish credible sources from those motivated by profit. Messages on social media are frequently designed to promote products, creating the impression that scientific information is straightforward when, in reality, it is often complex (10). Diviani (11) emphasizes that the ability to assess health information critically is arguably the most crucial component of HL moving forward. Health information appraisal encompasses a set of analytical skills and a robust knowledge base that enable individuals to effectively evaluate the credibility and relevance of health-related information (4, 12). This process involves discerning reliable sources from misleading or inaccurate claims. A Norwegian government report shows that more than half of children aged 9 use social media, and 86 percent of young people aged 9 to 18 are active on these platforms (13). This emphasizes the important role that digital communication plays in the lives of young people in Norway, while also reflecting wider global trends in how they interact and connect (14). As a result, there is a greater need for young people to evaluate both the content of information and its source critically. Yet, adolescents are particularly vulnerable as they tend to interpret information with optimism and trust, often lacking the skepticism needed to evaluate credibility (12). Research on adolescent HL shows a social gradient, with youth from lower socioeconomic backgrounds exhibiting lower HL than their more advantaged peers (15–17). When it comes to gender differences in HL, the evidence is mixed: some studies report slightly higher scores among girls (16, 17), while others find no substantial gender differences (18).

Norwegian policymakers (19) agree with researchers that adolescents need to develop the ability to critically assess health information. Additionally, related concepts such as critical thinking and ethical awareness, as well as health and life skills, are emphasized throughout the core curriculum for students in both elementary and secondary education in Norway (20). This highlights the vital role schools play in developing these capabilities in adolescents. However, there is limited evidence on the challenges adolescents face in managing health information in their daily lives, as well as on how they use this information to improve their own health and that of others. One reason for this may be the lack of an established short measurement tool for CHL. The concept of CHL is complex, making it difficult to develop concise and practical measurement instruments. This study aims to address this challenge.

We have previously developed and piloted the Critical Health Literacy for Adolescents Questionnaire (CHLA-Q) to capture a range of CHL competencies among students in Norwegian lower secondary schools. In line with established conceptual understandings (1, 4, 9) the questionnaire comprises three key domains: (A) information appraisal, (B) awareness of social determinants of health, and (C) citizenship for health and well-being within the school context. The information appraisal domain of the CHLA-Q consists of two factors related to the perceived difficulty of appraising health information and one factor assessing self-efficacy in applying appraisal strategies (12, 21). The development and validation process of the CHLA-Q has been thoroughly documented in previous publications (5, 12, 21). The scales are based on adolescents’ perceptions of the difficulty of various tasks related to health information appraisal. Self-assessments are grounded in social cognitive theory (22, 23), which emphasizes self-efficacy—the belief in one’s ability to perform tasks. Individuals with high self-efficacy tend to set higher goals and remain committed to them, viewing the subject as valuable (24). This is particularly relevant in health education, where the skills and knowledge needed for health can vary over time and across contexts. While performance-based indicators, such as scenario-based appraisal tasks and source verification exercises, can provide direct evidence of proficiency, they often have limitations due to task format, domain familiarity, and testing conditions. Performance-based indicators may not fully capture the motivational and contextual barriers that impact real-world behavior. In contrast, self-report scales can effectively measure perceived capability, willingness to engage, and metacognitive monitoring. These are key determinants of whether adolescents will attempt, persist in, and apply appraisal skills beyond the classroom. However, it is important to note that perceived difficulty does not equate to actual skill; factors such as confidence, prior experiences, and social desirability can all influence perceptions. Considering this, a brief, unidimensional self-report measure can provide a practical perspective on CHL among youth.

### 1.1 Aims

In the present study, we tested three unidimensional short versions of the CHLA-Q domain A (CHLA-QA) using Confirmatory factor analysis (CFA) to select the most parsimonious model that achieves acceptable fit, strong factor loadings, minimal local misfit, and adequate reliability. The second aim is to assess convergent and known-groups validity and measurement invariance across gender and grade level. To establish known-groups validity, we expect the scale to differentiate between groups based on background variables such as sex, age, parents’ education, number of books at home, general health literacy, and academic achievement. To examine convergent validity, we hypothesize moderate correlations with generic health literacy, subjective health, and health-related quality of life.

## 2 Materials and methods

### 2.1 Participants and procedure

The present study builds on cross-sectional data collected during the autumn of 2021 in the Literacies for Health and Life Skills Project (25). Five lower secondary schools participated in the study, with two located in rural areas and three in urban areas. Two schools had fewer than 200 students, one had approximately 300, and the remaining two had more than 450. In total, 1,592 students were enrolled during the study period. To participate in the study, students needed to provide both their own and their parents’ consent. The survey typically took 10 to 20 min to complete for most students. Those who did not receive consent to participate were assigned alternative tasks during the survey.

### 2.2 Measures

The data were previously used in the development (and validation) of a comprehensive measure of CHL, encompassing three domains and five factors (12, 21). In the questionnaire, Domain A focuses on individuals’ perceived ability to evaluate health information. This domain comprises two factors: perceived ability to assess the accuracy of health information (4 items) and perceived ability to determine the relevance of health information (3 items). These two short scales serve as the vantage point for constructing a shorter unidimensional scale in the present study. All items are scored on a five-point Likert scale (1: very difficult; 2: difficult; 3: sometimes difficult; 4: easy; 5: very easy), where respondents are asked how difficult it was for them to: (chl1) decide whether the media information about illness can be trusted, (chl2) decide whether information about healthy habits are also commercials, (chl3) decide whether the media information about healthy habits can be trusted, (chl4) find out whether health information is relevant to you when you are ill, (chl5) judge what food is healthy for you, (chl7) judge whether information on healthy habits is suitable for you and (chl8) judge whether information about unhealthy habits can be trusted. The item chl6 was excluded in the earlier scale development due to conceptual alignment and statistical performance (21).

*Health literacy* (HL) was evaluated using the Health Literacy among School-Aged Children (HLSAC) instrument (26). Developed in Finland, this instrument consists of 10 items. It has been tested in several European countries (27), and the Norwegian version has been used in previous studies in this age group (18). *Health-related quality of life* (HRQOL) was assessed using the Norwegian 10-item version of the KIDSCREEN-52 (28). *Subjective health* (SH) was assessed using a single question regarding participants’ health status (1: bad; 2: fairly good; 3: good; 4: very good; 5: excellent). SH is a well-documented predictor of future health outcomes (29).

Background variables measured included gender, grade, parents’ education (1: less than 3 years; 2: 3 years or more), and books at home (1: 0–10; 2: 11–50; 3: > 50). *Academic achievement* was measured using a single question about the grade students received in the subject during the previous semester’s assessment. In Norwegian schools, students are graded on a scale from 1 (fail) to 6 (best), indicating their achieved subject competence each semester. We examined which *sources of health information* students used most frequently through eight questions scored on a scale from 1 (very rarely) to 4 (very often). The students evaluated four different sources of health information (asking their parents, searching the internet, consulting healthcare professionals, and others) in two contexts: health promotion or disease prevention. In the results, we combined these dimensions to create a single indicator for each source of information.

### 2.3 Data analysis

All statistical analyses were conducted using RStudio (version 4.5.22025-10-31). The tidyverse packages (30) were used for data preparation, correlation, and item analyses. Factor analyses were performed using the lavaan package (31). Data loss was below 5% for all variables; in such cases, it is unlikely to introduce bias in the results (32). Consequently, predictive mean matching (PMM) was employed to impute the missing values, using information from other variables. Age (indicated by grade) and gender were included in the predictor matrices. The mice package (33) was used to inspect and impute missing data.

The first step in the data analysis was to perform an item analysis, examining the mean, skewness, range, and kurtosis for each item. Next, we assessed how each item correlated with the overall scale by calculating Spearman’s rho (*ρ*) between each item and the sum score of the other items. Additionally, we computed Spearman’s correlations between all items.

### 2.4 Item reduction and confirmatory factor analysis

We initially examined the extent to which a common factor could explain the variation within Domain A, which focuses on appraising health information. Based on findings from previous studies, we expected that the model would require adjustments. Utilizing modification indices, analyzing individual residuals, and assessing overall improvements in model fit guided our approach to streamlining the scale. While prioritizing statistical considerations, we also ensured that items from both factors in the original scales were retained. In the first step, we estimated a full-factor model that included all seven original items. Following our analyses, we first removed item chl1 and, in the final step, item chl3. The results from this final step are presented in detail in the results section, while more details on the first two models are available in the Supplementary materials.

Model fit was evaluated by examining standardized residuals. A close fit is indicated if the highest residual is less than or equal to 0.10, while less than or equal to 0.15 indicates an acceptable fit. Additionally, the unbiased standardized root mean residual (uSRMR) divided by the average communality (R^2^) should be at or below 0.05 for a close fit, and at or below 0.10 for an acceptable fit (34). Unlike traditional fit indices, such as the chi-square test for exact fit (χ^2^), comparative fit index (CFI), and root mean square error of approximation (RMSEA), this approach is unaffected by model complexity or sample size (34). Additionally, it accounts for the magnitude of factor loadings, thereby avoiding the reliability paradox, in which higher reliability can result in worse fit (34). Another advantage of this standardized method is its ease of interpretation across different studies (35). Consistent with prior research, we report recommended fit indices (χ^2^, CFI, and RMSEA) (32, 36) alongside uSRMR and uSRMR/R^2^. For this analysis, we used mean- and variance-adjusted unweighted least squares (ULSMV) estimation. This robust estimation method is preferred over the commonly used maximum likelihood estimator for non-normal and ordinal data (32, 36–38).

### 2.5 Measurement invariance

Measurement invariance was assessed across sex and grade using multiple-group CFA. We followed a recommended sequence for ordinal data (36), estimating: (1) a configural model with free factor loadings and item thresholds; (2) a threshold-invariant model with equal item thresholds across groups; and (3) a thresholds and loadings-invariant model constraining both thresholds and loadings. Invariance evidence was based on fit changes between nested models, accepting invariance if ΔCFI did not exceed 0.01 and ΔRMSEA did not exceed 0.015 (39). Aligning with the recommendations for assessing model fit in ordinal CFA models (34), we also monitored the uSRMR, with guiding cut-off values of ΔuSRMR ≤ 0.03 for the threshold step and ≤ 0.01 for the thresholds and loadings step (39).

### 2.6 Known-groups validity

To evaluate known-groups validity, we assessed whether CHLA-QA scores differentiated across relevant groups. Our primary comparison was three HLSAC-based health literacy categories: low (10–25), moderate (26–35), and high (36–40) (27). We expected that CHLA-QA scores would increase from low to high HL. Because scores were ordinal, group differences for variables with more than three categories (e.g., HLSAC, age categories, parental education, number of books at home) were tested using the Kruskal–Wallis H test. We reported epsilon-squared (ε^2^) as the omnibus effect size and interpreted 0.01, 0.06, and 0.14 as small, medium, and large, respectively (40). When the omnibus test was significant, we performed Holm-adjusted pairwise Wilcoxon rank-sum tests and reported the rank-based effect size r along with 95% confidence intervals. Effect sizes were interpreted as 0.10 for small effects, 0.30 for medium effects, and 0.50 for large effects (40). For dichotomous variables, such as gender, we used the Wilcoxon rank-sum test and reported the effect size r.

### 2.7 Correlation analysis and convergent validity

To evaluate whether the scale functioned as intended, we examined correlations with conceptually related constructs, including health literacy (HLSAC), health-related quality of life (HRQoL), and subjective health. We also explored correlations between CHLA-QA and academic achievement across several school subjects, as well as the frequency with which adolescents report using various sources of health information. We calculated Spearman’s rho (*ρ*) and applied a significance level of 0.05 to all analyses. Interpretations of correlation strength followed conventional guidelines: 0.1 to 0.29 is considered weak, 0.3 to 0.49 is moderate, and 0.5 or higher is strong (40, 41).

## 3 Results

### 3.1 Sample characteristics

The overall response rate for the primary survey was 33% (522 of 1,592) among eligible students from the five participating schools. Within individual classes, response rates ranged from 19 to 83%. An equal distribution of boys and girls from grades 8, 9, and 10 participated in the survey (Table 1). Most respondents reported having two parents born in Norway (70.1%) and spoke primarily Norwegian at home (77.6%). Only a small percentage (4.2%) indicated that they spoke a different language at home, while the rest varied between Norwegian and a second language (18%). Additionally, around 40% of the participants stated that both parents had completed more than three years of higher education.

**Table 1:** Participant characteristics.

Characteristics	*N* (%)
Gender	
Boys	248 (47.6)
Girls	273 (52.4)
Grade	
8th grade	164 (31.5)
9th grade	190 (36.5)
10th grade	167 (32.1)
Parents’ education	
Both > 3 years	311 (60.0)
One or both < 3 years/do not know	210 (40.3)
Books at home	
0–10	56 (10.7)
11–50	196 (37.6)
More than 50	269 (51.6)

### 3.2 CHLA-QA item analysis

All items showed a satisfactory distribution of responses, with a slight rightward skew. No questions exhibited severe (> ± 1) skewness, and all response options were used for all items. However, for question chl2, only one respondent selected category 1 (very difficult). Items 5 and 8 showed the greatest rightward skew and the highest mean (3.91 and 3.75), suggesting that respondents found them the least difficult. Both items 5 and 8 pertain to evaluating information about nutrition and other unhealthy habits, such as alcohol and tobacco use. In contrast, items 1, 3 (3.39 each), and 4 (3.37) had the lowest mean scores and showed no substantial skew, indicating that these items were perceived as the most difficult. Items chl1 and chl3 contain the word “media,” and item chl4 involves finding relevant health information when you are ill. Corrected item-scale correlations ranged from 0.47 to 0.60. We also calculated the Spearman correlation between all items. Most items were moderately correlated with each other (*ρ* from 0.2 to 0.5); one exception had a low correlation: items chl1 and chl5 (ρ = 0.0173).

### 3.3 Confirmatory factor analysis

We first estimated a one-factor model with all seven original items. This model did not show a good fit to our data, with fit indices falling outside the recommended values (χ^2^_mvadjusted_ = 226.5, *p* < 0.000. CFI = 0.90, RMSEA = 0.171 [0.152–0.191]. SRMR = 0.079, uSRMR = 0.054 [0.053–0.055]. uSRMR/ $\bar{R}$ ^2^ = 0.12). We also examined local misfit by analyzing the residuals and identified four exceeding the threshold for acceptable fit of 0.15: chl3 and chl1 (0.162), chl1 and chl5 (−0.176), chl3 and chl7 (−0.151) and chl5 and chl7 (0.202).

Items chl1 and chl3 are the only ones that contain the word “media.” We also observed that these items contribute substantially to the model’s local misfit. In the first step, we removed item chl1 before estimating the refined six-item factor model. Model fit was improved (χ^2^_mvadjusted_ = 125.5, *p* < 0.000. CFI = 0.93, RMSEA = 0.158 [0.134–0.183]. SRMR = 0.064, uSRMR = 0.042 [0.041–0.043]. uSRMR/ $\bar{R}$ ^2^ = 0.097), however, there was one individual residual above the threshold of acceptable fit, between items chl3 and chl2 (0.15). We continued by removing item chl3. The short-scale, now consisting of five items, showed positive results. Factor loadings ranged from 0.582 to 0.839 (Table 2). Additionally, the model fit indices indicate a good or acceptable overall fit (Model fit: χ^2^_mvadjusted_ = 29.3 (5), *p* < 0.000. CFI = 0.98, RMSEA = 0.097 [0.065–0.132]. SRMR = 0.037, uSRMR = 0.024 [0.021–0.026]. uSRMR/ $\bar{R}$ ^2^ = 0.055). We also assessed the local fit by examining the residuals between the empirical and model-implied correlation matrices, and we found no residuals exceeding 0.1. The Cronbach’s alpha for the scale is 0.79, which is considered a satisfactory level of reliability (42).

**Table 2:** Confirmatory factor analysis of one-factor short-version of the CHLA-Q.

Parameter	Unstandardized		Standardized		
	Estimate	SE	Estimate	SE	*p*-value
chl2	1.000		0.582	0.034	0.000
chl4	1.019	0.079	0.593	0.033	0.000
chl5	1.095	0.081	0.637	0.030	0.000
chl7	1.442	0.094	0.839	0.025	0.000
chl8	1.056	0.083	0.615	0.034	0.000

Model fit: χ^2^_mvadjusted_ = 29.3 (5), *p* < 0.000. CFI = 0.98, RMSEA = 0.097 [0.065–0.132]. SRMR = 0.037. uSRMR = 0.024 [0.021–0.026]. uSRMR/ $\bar{R}$ ^2^ = 0.055. Alpha_Ordinal_ = 0.79.

### 3.4 Measurment invariance

We evaluated configural, threshold, and thresholds-and-loadings invariance of the CHLA-QA across sex and grade level using multiple-group CFA. The configural model for gender groups showed acceptable incremental fit (CFI = 0.984, RMSEA = 0.085, uSRMR = 0.030). In the next step, we constrained thresholds to be equal, resulting in differences in fit indices within the recommended range (ΔRMSEA = − 0.070; ΔuSRMR = 0.007) and slightly above the recommended range (ΔCFI = 0.015). Imposing equality on both thresholds and factor loadings yielded differences within recommended bounds (ΔCFI = −0.002, ΔRMSEA = 0.009; ΔuSRMR = 0.006).

The configural model with groups based on grade level achieved acceptable incremental fit (CFI = 0.977, RMSEA = 0.101, uSRMR = 0.021), except for the RMSEA value. Constraining thresholds to be equal improved CFA and RMSEA considerably (ΔCFI = − 0.023, ΔRMSEA = −0.101, ΔuSRMR = 0.012). Adding equality constraints on loadings produced minimal changes in CFI and RMSEA within the recommended cutoffs (ΔCFI = 0.001, ΔRMSEA = 0.012). The increase in uSRMR in the final step was small but slightly above the 0.01 guideline for the thresholds-and-loadings invariance (ΔuSRMR = 0.011).

### 3.5 Known-groups validity

We examined whether CHLA-QA could detect the expected differences across groups (Table 3). In this study, girls had significantly higher scores than boys (*p* < 0.001), with a small effect size (r = 0.169). Grades had a significant effect on scores, with an overall effect size of small magnitude (ε^2^ = 0.013). Ninth graders scored significantly higher than eighth graders and higher than tenth graders, whereas there was no significant difference between eighth and tenth graders. Regarding parental education, adolescents with parents who had more than three years of education showed significantly higher scores, with a small effect size (r = 0.111). A similar pattern was observed for the number of books in the home, with a stepwise increase in CHLA-QA scores in homes with more books, with a small overall effect size (ε^2^ = 0.024). There was statistically significant difference between levels 1 (0–10 books) and 2 (11–50 books), and 1 and 3 (> 50 books), effect sizes were small (r_1-2_ = 0.160, r_1-3_ = 0.200). When we divided the sample into three levels (high, moderate, low) based on HLSAC scores, we again observed a stepwise increase: those with the lowest generic health literacy also scored lowest on CHLA-QA. Differences were statistically significant between all levels (*p* < 0.001). Overall effect size was moderate (ε^2^ = 0.132), and between-group effect sizes were small (r_mod-high_ = 0.289, r_mod-low_ = 0.246) to large (r_high-low_ = 0.654).

**Table 3:** Measurement invariance of the CHLA-QA across gender and grade level.

Characteristic	Invariance Model	CFI	RMSEA	uSRMR	ΔCFI	*Δ*RMSEA	ΔuSRMR
Sex	Configural (no constraints)	0.984	0.085	0.030	-	-	-
	Equal thresholds	0.999	0.015	0.037	0.015	−0.07	0.007
	Equal loadings & thresholds	0.997	0.024	0.043	−0.002	0.009	0.006
Grade	Configural (no constraints)	0.977	0.101	0.021	-	-	-
	Equal thresholds	1.000	0.000	0.033	−0.023	−0.101	0.012
	Equal loadings & thresholds	0.999	0.012	0.044	0.001	0.012	0.011

ΔDifference in the respective fit indices between nested models.

### 3.6 Correlation analysis and convergent validity

A moderate correlation was found between CHLA-QA and generic health literacy (*ρ* = 0.461) (Table 4). Additionally, moderate associations were observed with self-rated health (ρ = 0.316) and health-related quality of life (ρ = 0.346). Statistically significant but weak correlations were also identified between CHLA-QA and academic achievement in five of the six school subjects tested (Norwegian language arts, mathematics, science, physical education, and social studies). However, no significant correlation was found between CHLA-QA and the subject “Food and health”. Finally, we investigated whether CHLA-QA was correlated with the frequency with which adolescents reported using various sources of health information. A weak but significant correlation was observed between higher CHL scores and asking parents for help with health-related questions. For other sources, such as searching the internet, consulting healthcare professionals, or asking other individuals (e.g., teachers), no significant correlations were found (see Table 5).

**Table 4:** Known-groups validity for the CHLA-QA.

Characteristic	CHLA-QA	*p* value	Effect size
Gender		< 0.001***	r = 0.169 (CI: 0.09–0.26)
Boys (*n* = 248)	17.6 (3.0)		
Girls (*n* = 273)	18.7 (3.0)		
Grade		0.013*	ε^2^ = 0.013
8th grade (*n* = 164)	17.8 (3.2)	8th-9th*	r = 0.138 (CI: 0.03–0.24)
9th grade (*n* = 190)	18.6 (2.9)	9th-10th*	r = 0.128 (CI: 0.02–0.23)
10th grade (*n* = 167)	17.9 (3.0)	8th-10th NS	r = 0.018 (CI: 0.00–0.13)
Parents education		0.011*	r = 0.111 (CI: 0.02–0.20)
Both > 3 years (*n* = 311)	17.8 (3.1)		
One or both < 3 years/do not know (*n* = 210)	18.5 (2.9)		
Books at home		0.001**	ε^2^ = 0.024
1: 0–10 (*n* = 56)	16.9 (3.18)	1–2 *	r = 0.160 (CI: 0.03–0.28)
2: 11–50 (*n* = 196)	17.9 (2.76)	1–3 ***	r = 0.200 (CI: 0.09–0.30)
3: More than 50 (*n* = 269)	18.5 (3.15)	3–2 NS	r = 0.089 (CI: 0.01–0.19)
HLSAC		< 0.001***	ε^2^ = 0.132
1: High (*n* = 64)	20.7 (3.1)	1–2 ***	r = 0.289 (CI: 0.19–0.37)
2: Moderate (*n* = 402)	18.0 (2.6)	1–3 ***	r = 0.654 (CI: 0.55–0.74)
3: Low (*n* = 55)	15.8 (2.8)	3–2 ***	r = 0.246 (CI: 0.16–0.33)

The dependent variable in all analysis was the sum score of CHLA-QA (ranging from 5–25). Kruskal Wallis test was used for Grade and number of books at home. Wilcoxon rank-sum test was used for pairwise comparisons. *p* levels below 0.05 were considered significant. ε^2^ was used to measure overall effect size for variables with more than two groups and r between groups.

**Table 5:** Convergent validity.

	CHLA-QA	*p* value
Health literacy (HLSAC)	0.461	< 0.000
Subjective health	0.316	< 0.000
Health related quality of life	0.346	< 0.000
Academic achievement in school subjects		
Norwegian language arts	0.193	< 0.000
Mathematics	0.128	0.003
Science	0.191	< 0.000
Physical education	0.181	< 0.000
Social studies	0.200	< 0.000
Food and health	0.044	0.313
Where I get information about health		
Ask parents	0.233	< 0.000
Search internet	0.057	0.196
Ask health personnel	0.025	0.576
Others	0.058	0.184

Correlation between CHLA-QA, generic health literacy, health-related quality of life, subjective health, academic achievement across selected school subjects and sources for health information. Spearman correlations are calculated for all variables.

## 4 Discussion

In this article, we underscore CHL among adolescents as a key concern for public health and education. More research is needed to understand adolescents’ ability to critically evaluate health information and to assess what helps strengthen this competence. Reliable and precise measurement tools are essential for both purposes. Using a step-by-step factor analysis, we propose a condensed version of the CHLA-Q (12, 21) that focuses on the first domain of the broader CHL construct, specifically the ability to evaluate health information. Additionally, we have analyzed group differences on the shortened scale, CHLA-QA, and explored correlations with related concepts.

The initial one-factor model, including all seven items, showed poor fit, with global fit indices below recommended thresholds and several large residuals. This was an expected outcome given that the items were originally designed for a two-factor structure. The term “media” can be misleading because it encompasses many different nuances. It can include social media, news media, Google searches, or regular television or streaming platforms. Schools often teach students to be skeptical of information on social media. At the same time, adolescents may be encouraged to trust established news media. This lack of differentiation in these items may have caused problems. Removing item chl1, which contributed substantially to the local misfit, improved the overall model fit. The item included the term “media” but also focused on information about illnesses, which may be less relevant or more difficult for younger respondents, who often lack personal experience with serious health issues. Although the global fit improved, one individual residual between chl3 and chl2 exceeded the acceptable threshold, indicating the need for further refinement. The final step involved removing item chl3, yielding a five-item scale with strong psychometric properties. The confirmatory factor analysis indicated that the revised scale achieved a good model fit, with all fit indices falling within acceptable ranges. Factor loadings were significant and strong, and no residuals exceeded the local misfit threshold. Furthermore, the scale demonstrated satisfactory internal consistency, in line with established benchmarks for scale reliability (43).

The results from the invariance testing largely support approximate invariance across genders, despite a marginal ΔCFI at the threshold step; the thresholds-and-loadings step was within recommended ranges. Across grades, results are more mixed. The configural RMSEA was high; however, RMSEA can be unstable in ordinal factor models (35). As a result, changes in RMSEA between steps can be counterintuitive. Also, the uSRMR increased slightly beyond the 0.01 guideline at the final step for grade levels. Taken together, these findings suggest approximate invariance by sex but weaker support across grades.

The CHLA-QA demonstrated known-groups validity, distinguishing between groups with theoretically plausible differences. As demonstrated in other studies, socioeconomic status is linked to higher levels of HL (15–18), corresponding with our results. Adolescents whose parents had more than 3 years of education, as well as those from homes with more books, showed gradual increases in CHLA-QA scores. There were significant differences between the lowest and highest book categories, which reflect the influence of socioeconomic factors on CHL. Convergent evidence was also observed with levels of general health literacy, showing that CHLA-QA scores increased consistently across groups with low, moderate, and high generic health literacy (all *p* < 0.001). The effect sizes for these comparisons range from small to large, making them practically significant. The analysis revealed that ninth graders outperformed both eighth and tenth graders, with no significant difference in the scores between the eighth and tenth grades. This finding could be influenced by factors such as differences between the cohorts, variations in the curriculum, or differences in the sampling methods. Additionally, it may suggest a limitation in the scale’s ability to distinguish between age groups, which aligns with the mixed results from the invariance test across grades. A possible interpretation is that as adolescents grow older, their critical evaluation skills improve, but they also become more aware of the complexity of health information. Consequently, they may rate their abilities lower than they are. This issue requires further investigation and highlights a challenge for self-report measures in effectively capturing differences in CHL across age groups. In our study, girls scored higher than boys, a finding supported by some studies (16); others report no significant difference in HL levels (15, 18). Overall, the results support the CHLA-QA’s ability to differentiate between groups in conceptually sound ways.

The convergent validity analysis revealed relationships between CHL and several conceptually related variables. As anticipated, we observed positive moderate correlations between CHLA-QA and generic health literacy, self-rated health, and health-related quality of life. These findings align with existing research highlighting the connection between CHL, general HL, and overall health outcomes (44). Taken together, these findings suggest convergent validity for the CHLA-QA, although further investigation is warranted. Weak but statistically significant correlations were found between CHLA-QA and academic achievement across most subjects. This pattern indicates a modest relationship between CHL and overall academic performance, potentially reflecting shared cognitive or problem-solving demands. Kinnunen et al. (45) also found that general health literacy is associated with academic performance. However, health literacy did not mediate the negative relationship between academic performance and substance use. Given that adolescents’ decisions are strongly driven by socio-emotional factors (46), it is essential to develop health literacy alongside supportive socio-emotional environments that encourage sound, health-promoting decisions.

The lack of a significant correlation between CHLA-QA and the “Food and health” subject warrants further investigation. One possible explanation is that the curriculum content or pedagogical practices used in this subject may differ from those used in other disciplines, thereby reducing its alignment with the CHL construct (47). At the same time, the curriculum includes specific competence aims related to critically evaluating health and nutrition information, which means that the subject should, in principle, support CHL development. This discrepancy highlights the need to examine how CHL-related competencies are interpreted, taught, operationalized, and assessed within Food and Health.

We also examined the relationship between CHLA-QA and the frequency with which adolescents reported using various sources of health information. A weak but significant correlation was found between CHLA-QA and the tendency to ask parents for health-related information. This pattern may indicate that adolescents with stronger CHL are more inclined to involve trusted adults in health discussions, possibly because they are more aware of the value of reliable guidance or feel more confident initiating such conversations. The findings suggest that adolescents who feel supported by their parents in matters of health also express greater confidence in appraising health information and, in turn, experience less difficulty evaluating it. Findings from a qualitative study support the importance of parental support for adolescents in health information appraisal (48). They found that adolescents recognize strategies for assessing credibility and accuracy, such as comparing sources, checking authorship, and evaluating publication dates. However, knowing these strategies does not guarantee their effective application, as this often depends on individual and contextual factors. Consequently, adolescents may still feel overwhelmed and in need of support from caregivers or adults when processing health information. In line with a distributed health literacy perspective (49), the positive relationship between CHL and adolescents seeking help from their parents suggests that adolescents’ CHL involves the metacognitive skill of strategic help-seeking. In supportive environments, uncertainty may prompt active inquiry rather than inaction, encouraging adolescents to adjust their confidence levels, verify information, and rely on trusted expertise. Conversely, in situations where reliable adult support is less accessible, the same uncertainty can become more problematic, highlighting disparities in the availability and quality of support networks. We did not find significant correlations between CHL levels and the use of other information sources, such as the internet, healthcare professionals, or peers. This suggests that while parents are key sources of health information during adolescence, other sources may be used less often or in different contexts. The cross-sectional study design limits causal conclusions, as various factors affect both CHL and help-seeking behaviors. However, the patterns indicate that CHL is enhanced by social networks, highlighting the need for school-family partnerships and interventions to provide adolescents with reliable support beyond their homes.

### 4.1 Strengths and limitations

Participation in the study was contingent on participants’ willingness and required parental consent. This could have led to biased selection from the population, limiting the generalizability of our results. Our sample included roughly equal numbers of boys and girls, and an equal distribution of 8th-, 9th-, and 10th-graders, which is a strength. Our measure of academic achievement was the expected grades in specific subjects. While this approach may have its limitations about potential reporting bias, previous research has indicated that self-reported grades tend to correlate positively with actual grades, partly because past evaluations and optimistic predictions about future performance often influence self-assessments of grades (50). To examine our sample, we compared the mean expected grades of 10th graders with the national average (51). Across all subjects, the expected grades in our sample were slightly higher than the actual national grades. The differences in means ranged from 0.2 in social studies to 0.4 in mathematics and physical education. These differences may be due to overly optimistic expectations regarding grades. However, it is also possible that our sample is slightly skewed, with a higher proportion of high-achieving students compared to the overall population.

A key limitation concerns the removal of the media-related items (chl1, chl3). As noted in the discussion, the term “media” was semantically ambiguous in our context, which may have contributed to measurement problems. Nevertheless, given the centrality of social media–based information and misinformation to adolescents’ CHL, omitting these items may reduce content validity and conceptual coverage of the final instrument. Conceptually, appraising media information may warrant separate assessment; future studies should explicitly examine this dimension, potentially using or adapting targeted media-focused measures of health literacy (e.g., 52, 53). Our decision to reduce items was mainly based on data analysis, focusing on model fit and residual diagnostics. We also used the same sample used to develop the full CHLA questionnaire. This approach carries the risk of overfitting to the sample’s unique characteristics. Future research should aim to cross-validate the scale and its factor structure using independent samples. Also, we were unable to test the stability of CHLA-QA scores over time, and we remain uncertain about their sensitivity to genuine changes. Future research should evaluate test–retest reliability over meaningful intervals, establish longitudinal measurement invariance, and report appropriate stability metrics to differentiate between measurement noise and real changes.

Furthermore, this study relies on self-reports and indirect indicators, lacking external, performance-based measures. This limitation restricts our ability to assess objectively demonstrated competence, and research has shown that there are generally more measurement errors associated with self-reported indicators as opposed to performance-based measures (54). Future research should include performance tasks, such as evaluating health information and recognizing misinformation, to better distinguish between perceived appraisal ability and actual competence.

A strength of this study is that the scales are developed from a well-established theoretical framework of CHL (5, 12). Additionally, we conducted a thorough, step-by-step confirmatory factor analysis (CFA) and reported both traditional and newly developed fit indices. We ensured we examined both local misfit and global fit indices. We also tested the scale’s measurement invariance across gender and grade level. However, this study cannot definitively establish the invariance of the CHLA-QA; results are mixed across sex and grade, and replication in new samples is needed to confirm measurement properties. Future studies should consider partial invariance testing to pinpoint and accommodate localized lack of invariance.

## 5 Conclusion

We developed and validated a brief, unidimensional measure of adolescents’ information appraisal (CHLA-QA). Stepwise CFA yielded a parsimonious model with good fit and acceptable internal consistency. More research in independent representative samples is needed to establish the scale’s validity further. This study contributes preliminary evidence for validity, including expected known-groups differences by sex, grade, and socioeconomic markers, and moderate convergent correlations with generic health literacy, self-rated health, and health-related quality of life. Associations with academic achievement were weak but statistically significant across most subjects. The relationship between academic performance and CHL may reflect a common cognitive or problem-solving element. The positive link between higher CHL and help-seeking from parents is consistent with a distributed health literacy perspective, suggesting that efforts to strengthen health information appraisal should pair individual skill-building with school–family partnerships and equitable access to reliable guidance.

In future research, the CHLA-QA short form reduces respondent burden while retaining conceptual validity, making it suitable for surveillance and program evaluation. Limitations in this study include cross-sectional, self-report design and the single school setting. Although measurement invariance across gender and grade level was examined in this study, the results are inconclusive and warrant more research in independent samples. Future research should seek to establish measurement invariance, assess responsiveness to change over time, and cross-validate against performance-based indicators of CHL.

## Data Availability

A fully anonymized dataset can be made available upon request.
