# Supplementary material for: Exploring adolescents’ ability to appraise health information: validation of the self-report short-form Critical Health Literacy Questionnaire
Source: Front Public Health. 2026 Jul 9;14:1881570. doi: 10.3389/fpubh.2026.1881570 (PMC13391862; doi:10.3389/fpubh.2026.1881570)

Supplementary Material

1 Supplementary Data

The supplementary files contain detailed information from the analysis process. The first part involves item analysis and correlations for the original seven items. The second section presents the results from the CFAs of the two models that were not retained. At last, we present a figure showing the distribution of the final model's sum score for the CHLA-QA scale.

- Table S1. Item analysis for all the original items
- Table S2. Correlations between all items
- Table S3. Step one CFA. One-factor model of all seven original items.
- Table S4. Step two CFA. One-factor model of six original items
- Figure S1 Distribution of sumscore for the final short version of CHLA-QA

2 Supplementary Figures and Tables

Table S1. Item analysis for all the original items

Vars		Mean (SD)	Median	Skew	Kurtosis	ITC
chl1	decide whether the media information about illness can be trusted?	3.39 (0.77)	3	0.03	0.13	0.49
chl2	decide whether information about healthy habits are also commercials (healthy habits: physical exercise, nutrition etc.)	3.47 (0.81)	3	0.03	-0.39	0.56
chl3	decide whether the media information about healthy habits can be trusted?	3.39 (0.81)	3	-0.05	-0.13	0.59
chl4	find out whether health information is relevant to you when you are ill	3.37 (0.85)	3	-0.14	-0.23	0.51
chl5	judge what food is healthy for you	3.91 (0.91)	4	-0.71	0.30	0.47
chl7	judge whether information on healthy habits is suitable for you	3.61 (0.84)	4	-0.43	0.24	0.60

chl8	judge whether information about unhealthy habits can be trusted (unhealthy habits: alcohol, tobacco, nutrition etc.)	3.75 (0.94)	4	-0.67	0.45	0.52

Tabell S2 Correlations between all the original items

Items	chl1	chl2	chl3	chl4	chl5	chl7	chl8
chl1							
chl2	0.422						
chl3	0.534	0.526					
chl4	0.359	0.315	0.404				
chl5	*0.173	0.301	0.332	0.341			
chl7	0.321	0.392	0.326	0.439	0.510		
chl8	0.353	0.408	0.390	0.274	0.322	0.467	

* Spearman correlation below 0.2

Table S3. Step one CFA. One-factor model of all seven original items.

**Parameter**	**Unstandardized**		**Standardized**		
	**Estimate**	**SE**	**Estimate**	**SE**	**P-value**
chl1	1.000		0.674		.000
chl2	1.032	0.052	0.695		.000
chl3	1.109	0.052	0.747		.000

chl4	0.921	0.056	0.620		.000
chl5	0.799	0.055	0.538		.000
chl7	1.030	0.056	0.694		.000
chl8	0.929	0.053	0.626		.000

Model fit: $\chi^2_{mvadjusted}$ = 226.5, p < .000. CFI = 0.90, RMSEA = 0.171 [0.152 – 0.191]. SRMR = 0.079. uSRMR = 0.054 [0.053 – 0.055]. uSRMR/$\overline{R}^2$ = 0.12. $Alpha_{Ordinal}$ = 0.84.

Table S4. Step two CFA. One-factor model of six original items

Parameter	Unstandardized		Standardized		
	Estimate	SE	Estimate	SE	P-value
chl2	1.000		0.666		.000
chl3	1.003	0.049	0.668		.000
chl4	0.936	0.058	0.624		.000
chl5	0.911	0.056	0.607		.000
chl7	1.122	0.056	0.747		.000
chl8	0.934	0.059	0.622		.000

$\chi^2_{mvadjusted}$ = 125.5, p < .000. CFI = 0.93, RMSEA = 0.158 [0.134 – 0.183]. SRMR = 0.064, uSRMR = 0.042 [0.041 – 0.043]. uSRMR/$\overline{R}^2$ = 0.097). $Alpha_{Ordinal}$ = 0.82

2.1 Figure 1 Distribution of sumscore for the final short version of CHLA-QA

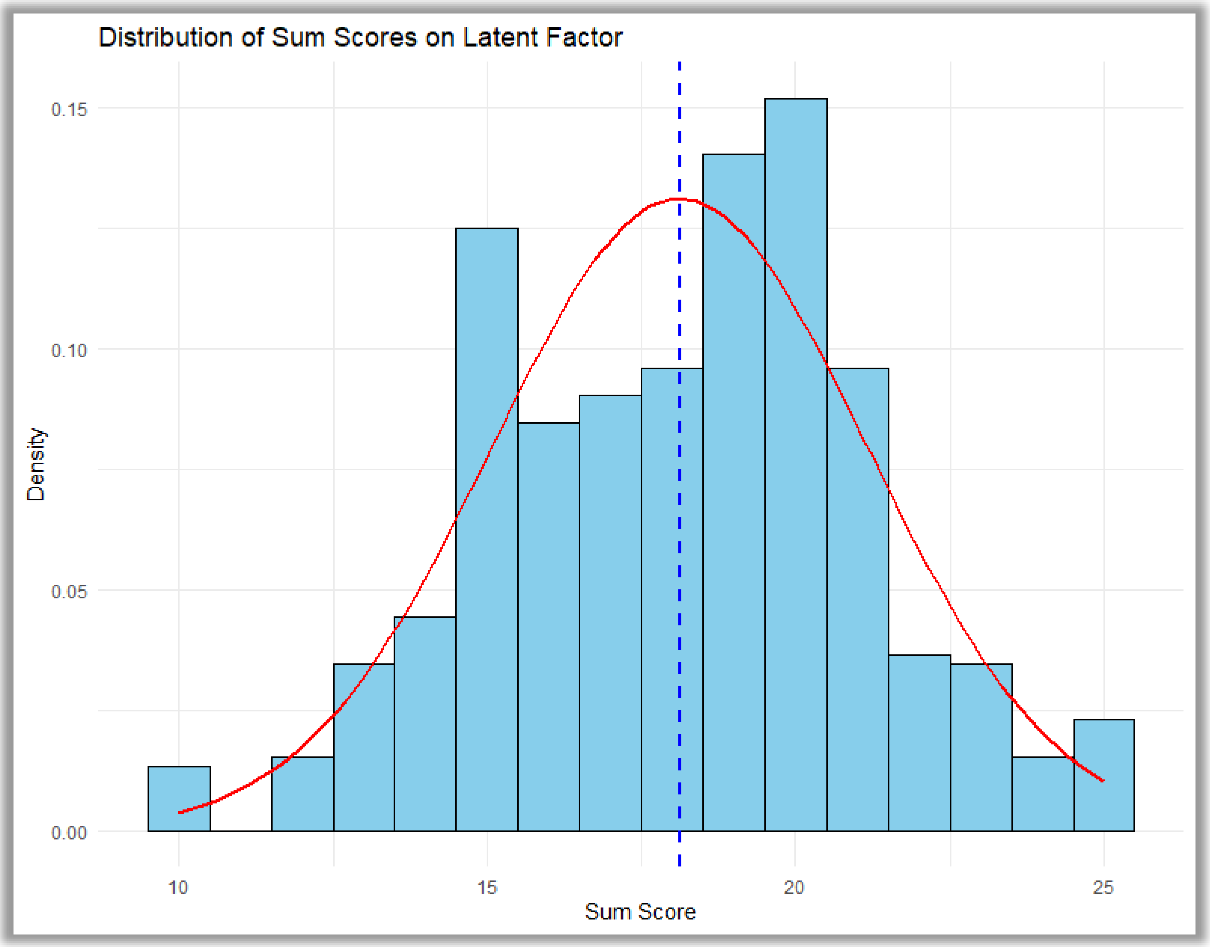

Supplement: Supplementary file 1 [file Data_Sheet_1.pdf]
